# Renal Parenchyma Perforation and Hematoma Formation following Double-J Stent Insertion in a Solitary Functioning Kidney: An Unusual Complication

**DOI:** 10.1155/2012/301275

**Published:** 2012-10-02

**Authors:** Michael S. Nomikos, Zacharias Chousianitis, Christos Georgiou, Chrysostomos Georgellis, Panagiotis Rikas, Theodoros Anagnostou

**Affiliations:** Department of Urology, Thriasio General Hospital, Genimmata Avenue, 19200 Athens, Greece

## Abstract

Double-J ureteral stent insertion is a common urological procedure performed for the relief of ureteral obstruction or as a part of other endourological procedures. Several complications have been reported in the past. A case of a 62-year-old woman who was stented due to hydronephrosis of her solitary functioning left kidney and had renal perforation and retroperitoneal hematoma formation is presented. She was managed conservatively with blood transfusion and double-J stent repositioning in the collecting system the fifth postoperative day. Follow-up noncontrast computed tomography (CT) of the abdomen was performed the first and third months after stent placement which showed stabilization of the hematoma.

## 1. Introduction

Ureteral stent insertion is a commonly used procedure in daily urology practice having been first described by Zimskind, for the treatment of ureteral obstruction and fistula [1]. Maturity of the technique paralleled development of extracorporeal shock wave lithotripsy and technical advances that allow treatment of various urological procedures [2]. The widespread use of ureteral stents has corresponded to the increase in possible complications related to stent insertion [3–5] (Table 1).

However, this is the first paper of renal parenchyma perforation and hematoma formation after an open end double-J stent insertion for the relief of ureteral obstruction in a solitary functioning kidney.

## 2. Case Presentation

A 62-year-old insulin-dependent diabetic female patient presented to our emergency department with pyrexia and left-sided renal colic. Her ultrasound kidney-ureter-bladder (KUB) examination revealed left-sided moderated hydronephrosis. A plain KUB film did not reveal any opacity at the course of the left ureter. The laboratory results were urea 64 mg/dL, creatinine 1.5 mg/dL, glucose 334 mg/dL, hemoglobin 11.6 g/dL, and white blood cells (WBC) 15.5 × 10^3^. She underwent noncontrast CT of the abdomen which revealed one stone at the level of 05 vertebrae (7 mm) and a second one at the level of 03 vertebrae (6 mm) causing left-sided pelvicalyceal dilatation. She was then admitted and commenced on antibiotics. The next day under local anesthetic, she underwent placement of an open end 4.8FR-26 cm D-J stent (Cook ureteral stent) under fluoroscopy. A hydrophilic guide wire (Road Runner straight tip, Cook) was used for stent placement. On the first postoperative day, she developed gross hematuria and left-side abdominal tenderness, with a total urinary output of 430 cc. Hemoglobin level dropped to 7.6 g/dL and creatinine raised to 1.8 mg/dL. She then underwent a noncontrast CT of the abdomen which showed a large perinephric collection located inferior and posterior to the left kidney.

**Figure 1:**
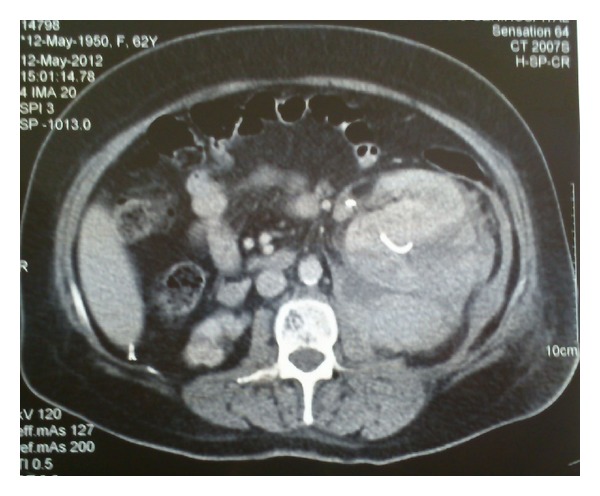
CT of the abdomen with i.v. contrast showing a 12 × 8 perirenal hematoma pushing anteriorly the left kidney with the tip of the stent penetrating the renal parenchyma.

**Figure 2:**
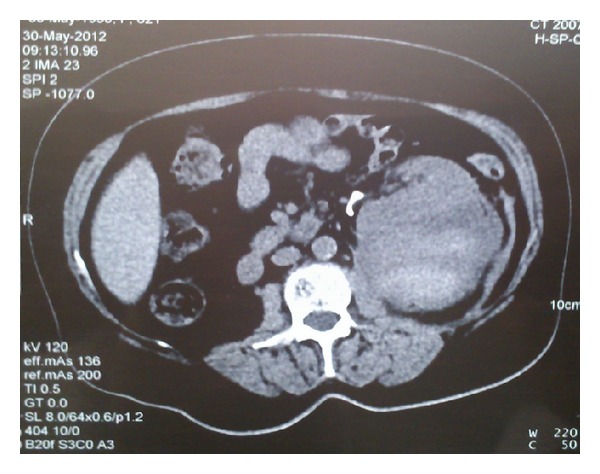
CT of the abdomen 3 weeks after stent placement showing stabilization of the perirenal hematoma with the D-J stent repositioned in the renal pelvis.

The patient was put to bed rest; 3 units of erythrocyte suspension and 2 units of fresh frozen plasma were administered. She was hemodynamically stable and urine cleared the third postoperative day. Diuresis was accomplished and creatinine levels normalized. The third postoperative day she underwent a second CT of the abdomen with intravenous contrast which showed a 12 × 8 stable perirenal hematoma pushing forward and medially the left kidney with the tip of the stent penetrating the renal parenchyma with no contrast extravasation from the collecting system of the kidney (Figure 1). The fifth postoperative day the stent was repositioned in the renal pelvis under fluoroscopy. She was discharged the ninth postoperative day. Follow-up CT of the abdomen the first month after stent placement showed stabilization of the hematoma (Figure 2).

## 3. Discussion

Ureteral stenting is a standard procedure in daily urological practice; however, significant complications may occur. We present a unique case of ureteral stent malposition with renal parenchyma perforation and hematoma formation in a solitary functioning kidney with hydronephrosis due to ureteral lithiasis. The unusual event of this case is that renal perforation happened after placement of an open end 4,8Ch D-J stent over a hydrophilic guide wire with a 3 cm soft straight tip which is considered to be impossible to penetrate the renal parenchyma.

Placement of the stent was relatively easy with no signs of extravasation from the pelvicalyceal system or renal perforation during the procedure. The most possible explanation for development of hematoma could be trauma to pelvicalyceal system during guide wire manipulation or raised intrarenal pressure leading to forniceal rupture, separation of capsule from parenchyma, and hematoma formation which should be the most possible explanation due to presence of infection and hydronephrosis in the same kidney.

Clinical presentation varies considerably with acute onset of flank and abdominal pain together with macroscopic hematuria being the most common symptoms. Treatment of these conditions has been debated. We successfully managed this patient conservatively since the majority of these hematomas resolve spontaneously. Indications for early intervention by means of percutaneous drainage could be done in patients with unbearable pain, infective complication of the hematoma itself and, renal compression and ischaemia if the hematoma is subcapsular and compresses the kidney. In case of uncontrolled bleeding or if the hematoma is rapidly progressive, conventional drainage by open surgery may be performed [6, 7]. Secondary infection is not an unusual event during resolution of the hematoma, making long-term antibiotic prophylaxis a good treatment option to prevent such a complication.

Followup should be individualized according to size of the hematoma and patient characteristics. Computed tomography of the abdomen should be performed the first and third month after hematoma formation with a renal ultrasound every 6 months thereafter.

Although a common procedure, ureteral stent placement is not without complications and a high clinical suspicion index is required for early diagnosis and proper management of poststenting renal hematomas. Close followup is very important to prevent hematoma-related complications.

## Figures and Tables

**Table 1:** 

Consequences and complications of ureteral stent placement
Irritative voiding symptoms
Incontinence
Suprapubic or flank pain
Vesicorenal reflux
Hematuria
Pyuria
Urinary tract infection
Malposition
Migration
Inadequate relief of obstruction
Encrustation
Ureteral erosion or fistulization
Fracture
Forgotten stent
